# Relationship between Ear Temperature, Behaviour and Stress Hormones in Guinea Pigs (*Cavia porcellus*) during Different Interactive Activities in Zoos

**DOI:** 10.3390/ani14071111

**Published:** 2024-04-04

**Authors:** Misako Namiki, Toshiharu Fukayama, Takane Suzuki, Ayumi Masaiwa

**Affiliations:** 1 Department of Animal Sciences, Teikyo University of Science, Tokyo West Campus, 2525 Yatsusawa, Uenohara City 409-0193, Japan; 2 Department of Animal Sciences, Teikyo University of Science, Senju Campus, 2-2-1 Senju Sakuragi, Adachi-ku, Tokyo 120-0045, Japan; t-fukayama@ntu.ac.jp; 3 Adachi Park of Living Thing, 2-17-1 Hokima, Adachi-ku, Tokyo 121-0064, Japan

**Keywords:** zoo animal welfare, animal visitor interaction, stress assessment, cortisol salivary, comparison between activities

## Abstract

**Simple Summary:**

At zoos, paying attention to animal welfare during interactive activities between exhibited animals and visitors is becoming increasingly necessary. Many zoos in Japan conduct petting activities where people place guinea pigs on their laps, but it is necessary to encourage participants to be aware of the welfare of the animals. The experience of involvement among the participants are involved in together plays an important role in promoting animal welfare. This study showed that changes in body temperature measured with a digital ear thermometer for human infants may be used as a physiological indicator. In addition, by simultaneously measuring the expression of negative behavior and stress hormones in guinea pigs and correlating it with changes in body temperature, it is possible to determine which types of interaction are or are not burdensome to guinea pigs. However, more data are needed to clarify the relationships between behavior, body temperature changes, and stress hormones.

**Abstract:**

Guinea pigs (*Cavia porcellus*) are used for interactive activities in zoos; therefore, it is important to investigate their welfare. This study aimed to evaluate the validity of measuring the guinea pigs’ body temperature of guinea pigs through the ear canal and investigate the relationship among changes in the expression of negative behavior, changes in body temperature, and changes in salivary cortisol concentration, and examine the effects of different interactive activities. In the normal interactive activities performed at the site, the decreased body temperature of pigs was observed over time. In contrast, increased body temperature was observed in excessive interactive activities, which are not recommended. Among the negative behaviors, “Head turning” and “Locomotion” increased significantly in excessive interactions compared to normal interactions, but “Head tossing” decreased significantly over time in both types of interactions. “Freezing” was observed only in excessive interactions. Salivary cortisol concentration increased significantly for all activities. Investigating the relationship between the individual expression of negative behavior and changes in body temperature and changes cortisol level made it possible to uncover the potential for inferring an animal’s physiological state. Combining ear temperature monitoring and behavioral observation during zoo interaction activities is recommended as an ethical and scientifically supported practice.

## 1. Introduction

Guinea pigs (*Cavia porcellus*) are commonly used in animal-assisted interventions and zoo interactive activities [1]. This is due to the fact that they rarely attack humans, and the ability to observe them up close leads to an even greater familiarity with them [2]. However, one challenge in using this species is assessing its condition [3], and an animal visitor interaction protocol (AVIP), including that assessment is recommended [4].

Regarding the welfare of Guinea pigs, indicators such as changes in fecal glucocorticoid levels and cortisol concentrations in the saliva have been used to confirm the presence of stress [5,6] or estimate the effects on social stress reduction by the diet content with reference to cortisol concentrations [7]. Moreover, estimates based on the frequency and duration of negative behavior have been used to assess the welfare of Guinea pigs [8]. Negative behaviour is indeed a survival behaviour, including risk avoidance, but depending on its increased frequency, it can lead to stress hormone exudation, and continued exudation can reduce the quality of life. Since salivary cortisol concentration measurement is time-consuming, it is important to establish a method that allows for real-time assessment during interactive activities and identifies which behaviors to focus on. A recent study investigated the correlation among changes in the eye temperature of guinea pigs during animal-assisted therapy or while observing for various types of negative behaviors [9]. Monitoring the welfare status of domestic animals in real-time by measuring their body temperature using a thermal camera and correlating it with certain behaviors is possible [10]. Stress in mammals and humans generally increases deep body temperature [11]. However, taking individual eye temperature measurements using thermal cameras for guinea pigs during interactive activities in Japanese zoos is challenging due to the possibility of overlap between the human and animal measurements. In the present study, we first examined the validity of ear thermometers designed for human infants as an alternative method. Secondly, we investigated the impact of differences between two types of interactive activities using the following three indicators: changes in body temperature during interaction, expression of negative behavior, and cortisol concentration. The two different types of interactive activities between people and animals were the normal interactive activities recommended by Adachi Park of Living Things (site of the experiment), which do not place a heavy load on animals, and excessive interactive activities, in which a moderate load is placed on animals.

## 2. Materials and Methods

### 2.1. Subjects

**Figure 1:**
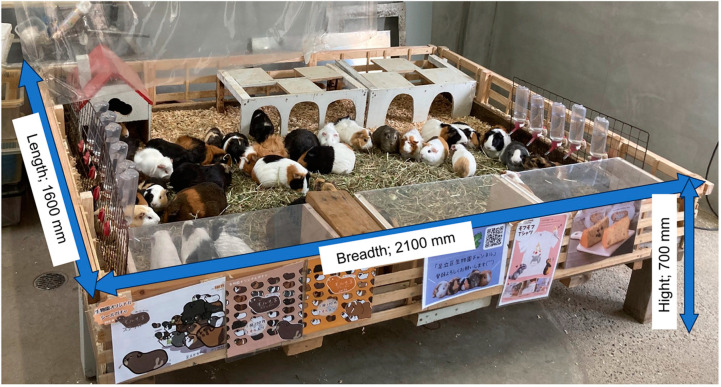
Guinea pig enclosure at the experiment site. Floor area of 1600 × 2100 mm, height of 700 mm, equipped with shelters. Hay (Timothy), pellets, and water were freely accessible for the animals. The age ranged from 5 months to 30 months (mean: 16, S.D. = 8.2), and the body weight ranged from 624 g to 1082 g (mean: 891.2, S.D. = 150.1).

Of the 41 female guinea pigs housed in the animal housing facility at Adachi Park for Living Things (housing density: 12.2 guinea pigs per m^2^), 19 pigs aged 3–24 months were included in this study. The pigs that met the age requirements for participation in interactive activities (at least 3 months) and those who had passed a minimum of 3 months post birth were selected. Nineteen individuals who had never previously participated in an interactive activity at the zoo to which the co-researcher belongs (Adachi Park) were selected. In fact, this is to provide information on how to advise activity participants on interacting with guinea pigs following the post-Corona park opening. The subjects had no experience of being on the lap of the participants of the interactive activities. However, from 18 October 2022 to 3 November 2022, they were briefly patted lightly on the back without being placed on the lap. Furthermore, the staff held and moved all animals regularly during the daily care and body weight measurement (Figure 1).

### 2.2. Measurement of Rectal and Ear Temperatures

Guinea pigs possess an external auditory canal that bears a striking resemblance to that of humans and features a linear structure leading to the eardrum [12]. This observation, coupled with the assertion that guinea pigs are a more suitable model for human inner ear anatomy than rats due to this similarity [13], allowed us to hypothesize that it may be possible to use a human infrared ear thermometer to measure the ear temperature of guinea pigs. If a correlation between rectal and ear temperatures is observed, ear temperature can be used instead of rectal temperature to determine the core body temperature. To confirm this, the right ear canal temperature was measured once per animal at the same time as the rectal temperature was measured. The rectal and ear temperature measurements were taken on 12 September 2022, between 1010 h and 1040 h. The atmospheric temperature was 28.2 °C at the beginning and 28.8 °C at the end of the measurement. Rectal temperature was measured by an experienced veterinarian using a digital veterinary thermometer (C.I. Medical Co., Ltd., Genia, Chaléons, France, https://vet.feed.jp/product/500136410.html (accessed on 28 March 2024)). (Measurement range, 32–42.9 °C; measurement accuracy, ±0.1 °C; power source, LR41 1.5 V battery; medical device thermometer certified under ASTM prEN12470-3:1997; https://www.medicalexpo.com/ja/prod/genia/product-80292-951919.html, accessed on 28 March 2024). During rectal temperature measurement, a researcher inserted a human infrared ear thermometer (weight, 52 g; dimensions, 96.3 mm wide × 24.5 mm long × 51.3 mm high; range of measurement, 34.0–43.0 °C; power source, CR2032 lithium battery [Medical Device Approval No. 302AFBZX00062000]; measurement site, forehead or ear, HuBDIC, Tokyo, Japan) into the ear canal of each animal and measured its body temperature. The rectal thermometer was inserted for less than 10 s, whereas the time taken to open up the auricle and finish measuring the temperature was approximately 5 s. Moreover, the restraint time per individual was under 30 s, and if it exceeded that, the measurement was terminated. As a result, ear temperature measurement was not possible for 1 individual out of the 19, leaving 18 individuals for analysis.

### 2.3. Experimental Design of Monitering Interactive Behavior, Ear Temepreture Mesurement and Colletion of Salivery

**Figure 2:**
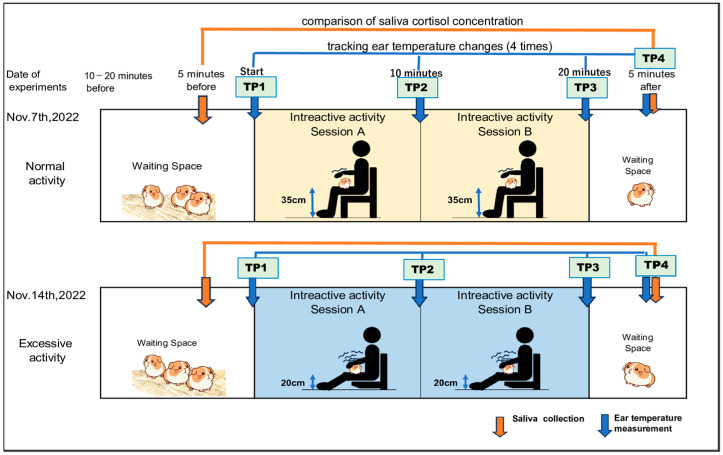
Experiment Design: The timing of saliva collection and body temperature measurement and the way of interactivity. “TP” means the time point when ear temperature mesuerment is achieved.

Subjects were moved from their housing facility to a holding pen 10–20 min prior to interaction activities. One session lasted for 20 min. Saliva samples were collected (orange arrows) for 5 min. before and 5 min. after, and ear temperature was measured (blue arrows) four times: [TP1], [TP2], [TP3], and [TP4]. The interval between the two sessions was 1 week in both the normal (top) and escessive methods (bottom) (Figure 2).

### 2.4. Replication of Different Interaction Methods and Setting of Interaction Duration

In this experiment, the interactive activity between a guinea pig and a person took place in the following order:

(1) A handler moved the animals out of their enclosure and transferred them to a crate 10–15 min before the start of the interactive activity, which was placed near the site of the interactive activity.
(2) A towel was placed on the lap of each participating research assistant who was seated and waiting.
(3) A staff member picked up one animal at a time, held it in their arms, and then placed it on the handler’s lap. After the interactive activity, the care staff moved the animals back to the crate and returned them to their enclosure. During the normal interactive activities, the handlers were instructed to pet the animal gently on their lap. For excessive interactive activities, they were instructed to randomly perform the following actions 2–3 times a min on their lap: holding the animal, patting the head, stroking the reverse, and touching the abdomen. With either of the interactive activity methods, when the animal tends to climb down from the lap to the floor, a staff member would put their hand against the animal and keep an eye on it until it was safe. If it climbed down onto the floor, the staff member would pick it up and hold it in their arms before returning it to the lap of the handlers.

All of the handlers were university students (Department of Animal Sciences at Teikyo University of Science) specializing in animal care, with 3 women in normal and men and 5 women in excessive. In the order of number on the GPs list, the care staff was responsible for each handler, with one animal on their lap for 20 min of interaction. One researcher took saliva samples from all individuals on her lap, another researcher took body temperature measurements.

The normal interactive activity occurred on 7 November 2022, from 1015 h to 1305 h, whereas the excessive interactive activity occurred on 14 November 2022, from 1005 h to 1215 h. The care staff, who managed the animals’ safety, handled, observed, fed, and watered them while they were in the crate.

### 2.5. Monitoring the Expression of Negative Behavior

A video camera mounted on a tripod was placed approximately 5 m away from the interaction site, and the recorded data were used to analyze the occurrence of negative behaviors. The ethogram was based on that used by Gut et al. [14] and Cohen and Beths [15] and included the following five behaviors observed on the lap of research assistants: “Head tossing”, “Locomotion”, “Head turning”, self-grooming by nibbling or licking their fur (“Licking”), and “Freezing”. For the “Freezing” behavior only, the duration was calculated in s. and the percentage of duration in the total observable time was used. In contrast, for the other behaviors, the frequency of occurrence per 60 s was used (total observation time was determined by subtracting the time during which observations were not possible due to ear temperature measurement or being out of sight, and the count of occurrences within that total observation time was converted to occurrences per 60 s). The detailed definitions and units of analysis for these negative behaviors are shown in Table 1.

Preliminary observations were used to identify these “negative behaviors”, and the video data analysts knew the behavior. The animals were assigned to handlers according to the age-ordered list.

### 2.6. Measurement of Ear Temperature during Interactive Activities

Ear temperature measurements were performed by the author or animal welfare staff on 7 November 2022, under normal conditions and on 14 November 2022, under excessive conditions for all subjects in the following four instances: (1) before interaction (activity start: time point [TP1]), (2) 10 min after the start of the interaction (time point [TP2]), (3) at the end of the interaction (activity finish: time point [TP3]), and (4) 5 min after the activity (time point [TP4]). Animals were moved by animal care staff at all times. During ear temperature measurement, the researcher or care staff held the animal in her lap, with the animal’s abdomen down.

### 2.7. Measurement of Salivary Cortisol Concentration

Salivary was collected before 5 min of activity and after 5 min activity, and the amount of change between the two was used as a marker. Cortisol exudation time from the adrenal gland to the blood is very rapid, but exudation to the salivary glands can be considered to be weight and species-specific for that animal [18]. The exact data on exudation time to the salivary glands in GP is not clear in the literature, but in the authors’ prior study, the highest levels of salivary gland exudation were observed at 5 min after the interaction activity, and returned closer to the levels before the activity after 20 min, so it was judged appropriate to collect samples 5 min after the end of the activity in this study. The researcher held the animal on her lap, inserted a cotton bud into its mouth about 20–30 s, took a saliva sample (about 0.1–0.2 mL each), residue removed via centrifugation, and measured the cortisol concentration using a DetectX Cortisol ELISA Kit (Arbor Assays, Ann Arbor, MI, USA). We have been measuring salivary cortisol levels as a non-invasive stress measurement method during guinea pigs’ participation in interactive activities in several zoos in Japan for pre- and post-activity comparisons, using the same kits, sample handling, and analysis methods.

Salivary cortisol concentration was measured according to the instructions attached to the measurement kit. After the sample was about 2 h of refrigerated storage, removed residues were by 2000× *g* 20 min, centrifuged (4 °C, 13,000× *g*, 10 min), and stored at −78 °C. The measurement procedure was after thawing the samples at room temperature; the dilution rate was 15 times using Assay Buffer (part of the kit, Ann Arbor, MI, USA), then using wavelength 450 nm absorbance by plate reader (Molecular Devices, spectraMax Plus384, San Jose, CA, USA). The average value between the two wells of the plate was used for any sample. The quantitative range for cortisol measurement was 750 to 48,000 pg/mL. The intra-assay CVs were 11.4 (Before) and 11.5 (After) in Normal, 8.0 (Before) and 8.58 (After) in Excessive. Inter-assay CVs were 9.7 (Before)and 10.0 (After).

### 2.8. Analysis

Changes in body temperature were evaluated individually for each subject by measuring the body temperature. The average value (unit: °C) for each measurement was calculated. Subsequently, the presence of significant differences in temperature was determined for the different time elapsed each time points ([TP1] and [TP2], [TP2] and [TP3], [TP3] and [TP4], [TP1] and [TP4]), both the normal (n = 19) and excessive (n = 19) interactive activities, using *t*-test (Corresponding two-sample *t*-test to examine the difference between the each time-specific differences). A one-way analysis of variance (ANOVA, type I, to determine significant differences between data groups at four time points) was used to assess the overall change.

Negative behavior was analyzed for each subject individually. The expression frequency per 60 s was calculated for the following behavior categories: “Head tossing”, “Head turning”, “Locomotion”, and “Licking” in both the Sessions A and B. The expression frequencies were due to the fact that the video data available from the normal interactive activity included 12 individuals, whereas the number of individuals differed between the normal (n = 12) and that from the excessive (n = 19) interactive activities. Wilcoxon signed-rank test was performed to compare the expression frequencies between the normal and excessive interactive activities. The comparison between the two Session for each behavior category was analyzed using a corresponding two-sample *t*-test. In both cases, there were periods of non-observation, ranging from a few s to 160 s, due to moments when temperature measurement was taking place or when the guinea pig was not visible on camera. To account for these non-observed periods, the total count of behaviors was determined by subtracting the non-observed time from the 20-min observation period and then calculating the frequency per observation time (60 s). Furthermore, for the percentage of duration in the total observable time was used for “Freezing” category.

Changes in salivary cortisol concentrations were analyzed for 18 of the 19 subjects, as measurements were not obtainable for one subject due to insufficient saliva volume. For both the normal (n = 18) and excessive (n = 18) interactive activities, the differences in cortisol concentration between 5 min before and 5 min after the interactive activity were assessed using corresponding two-sample *t*-tests, all data for saliva cortisol concentrations were subjected to a natural logarithmic transformation (LN) followed by the *t*-test.

Pearson’s correlation coefficient was used to examine each indicator of the correlation between temperature change and the frequency of each behavior. Furthermore, for individuals in whom “Freezing” was identified, the correlation between body temperature change and cortisol concentration change was confirmed by Pearson’s correlation coefficient.

### 2.9. Research Ethics Review

This study was performed in accordance with the Teikyo University of Science Guidelines for Animal Experimentation [20] and was performed after an ethical review by Adachi Park for Living Things, i.e., where the public zoo is located in Tokyo [Approval Dates: 2 September 2022 (Document No. 43), and 1 November 2022 (Document No. 50)]. During the review process, the authors decided that rectal temperature measurements would be performed by a veterinarian, ear temperature measurements and saliva collection would be performed by an experienced researcher, and interactive activities would be conducted by senior students majoring in animal science who had a complete understanding of the experiment’s objectives (fourth-year students at the time of the experiment). Endpoints were determined by either a veterinarian or guinea pig handlers. The research team consisted of the author, four staff members of Adachi Park for Living Things, one veterinarian, and six research assistants. The experiment was conducted exclusively within the zoo premises.

## 3. Results

### 3.1. Relationship between Rectal Temperature and Ear Temperature

**Figure 3:**
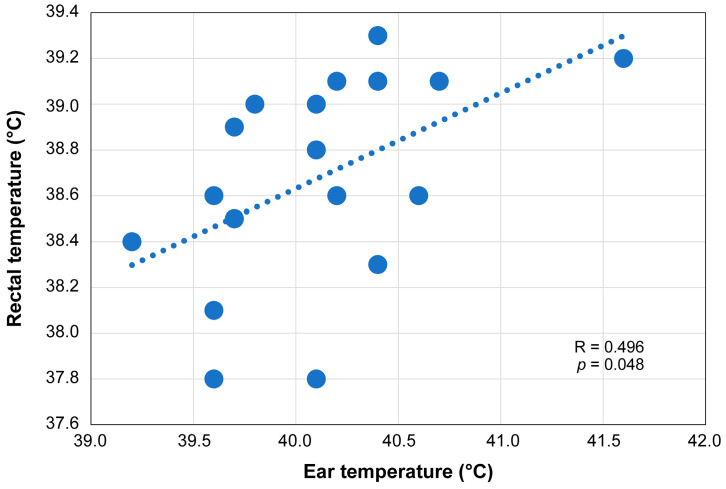
Rectal and ear temperatures of the guinea pigs.

Of the 19 guinea pigs, ear temperature measurement was performed for one (Azu: individual name); however, the duration of restraint exceeded 30 s, and consequently, the rectal temperature measurement was discontinued. Thus, the analysis was performed with 18 guinea pigs. Rectal temperatures ranged 37.8–39.6 °C (mean, 38.7 °C), whereas ear temperatures ranged 39.6–41.6 °C (mean, 40.1 °C). In all pigs, ear temperatures were higher than rectal temperatures. The correlation coefficient for both ear and rectal temperatures was 0.496 (*p* = 0.048) (Figure 3). Furthermore, the ear temperature of Azu was 40.1 °C, and although not measured simultaneously, the rectal temperature measured again after 35 min was 39.2 °C. Therefore, during the following interaction sessions, Azu was subsequently included as a subject for ear temperature measurement, behavioral observation, and saliva collection.

### 3.2. Changes in Body Temperature

**Figure 4:**
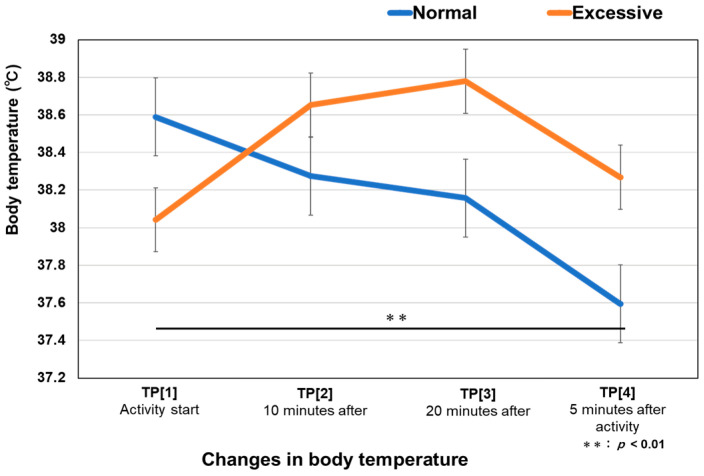
Changes in body temperature in two different activities. Error bars denote one standard error of the mean. TP is the temperature measurement point defined in the experimental design (Figure 2).

In the normal interactive activity (n = 19), the average temperature of [TP1] was 38.6 °C. It started to decrease after 10 min and continued to decrease until the end. In the excessive interactive activity (n = 19), the average temperature of [TP1] was 38.0 °C, which was lower than that observed in the normal interactive activity. After 10 min, it increased to 38.7 °C, reached 38.8 °C at [TP3], and then decreased to 38.3 °C at [TP4]. No significant difference between the normal and excessive interactive activities (df = 3, variance = 0.794, *p* = 0.720). However, a significant difference was observed in the normal interactive activity between [TP1] and [TP4] (t = 1.734, df = 18, *p* = 0.008) (Figure 4). In the normal interactive activity, the temperature gradually decreased, and the difference between [TP1] and [TP4] was significant. In the excessive interactive activity, the temperature gradually increased, but no significant difference was observed between any time points.

### 3.3. Behavioral Changes (Differences in Frequency of Occurrence over Time)

**Figure 5:**
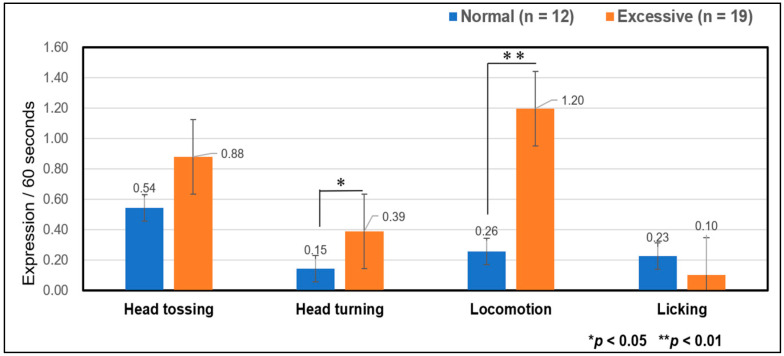
Comparison of the expression frequency of negative behaviors between normal and excessive interactive activities. Comparison between the normal (blue, n = 12) and excessive (orange, n = 19) interactive activities. Error bars denote one standard error of the mean.

In comparisons two different interactive activities, “Head turning” and “Locomotion” were expressed more significantly during excessive than during normal. No significant differences in “Head tossing” and “Licking” were observed between the two interactive activities, as confirmed using (Mann–Whitney U test: ”Head turning”; z = 2.447, variance = 601, *p* = 0.014, “Locomotion”; z = 3.417, variance = 607, *p* = 0.001) (Figure 5).

**Figure 6:**
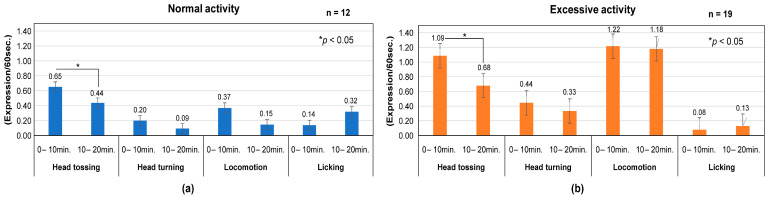
Negative behavior expression frequency in normal (**a**) and excessive (**b**) interactive activities (comparison between Session A and Session B. Error bars denote one standard error of the mean.

In comparison between sessions A and B within each activity, the frequency of “Head tossing” in the normal activity was significantly lower (t = 1.78, df = 11, *p* = 0.020) during Session B. Still, no differences were observed for other behaviors. In the excessive interactive activity, “Locomotion” consistently exhibited a higher mean frequency than the other negative behaviours, even as time passed (Figure 6).

**Figure 7:**
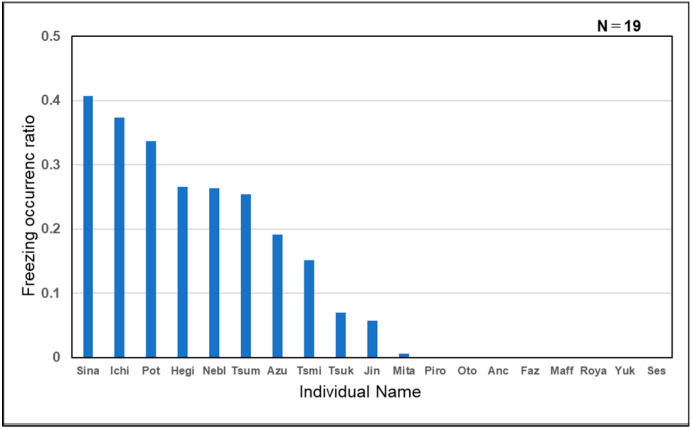
“Freezing” behavior occurrence ratio for each guinea pig (total: 11/19).

“Freezing” was observed only during the excessive interactive activity (11/19 individuals), with a mean duration of 244 s ranging from 6 s to 464 s, and the percentage of occurrence was represented (Figure 7).

### 3.4. Changes in Salivary Cortisol Concentration

**Figure 8:**
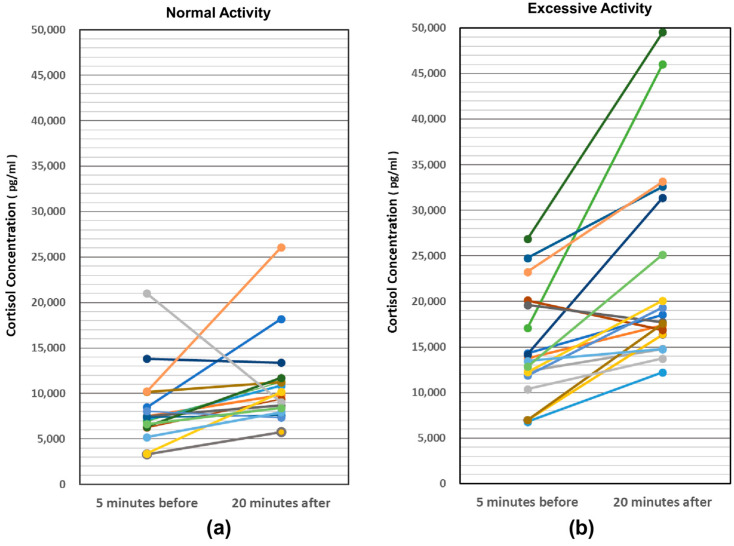
Changes in saliva cortisol concentration during normal and excessive interactive activities. Normal interactive activities (**a**) and Excessive interactive activities (**b**).

The saliva collected from 18 individuals was about 100–200 μL for both activities. One subject (Piro: individual name) had less than 30 μL in any activity. Since the sample from one of the 19 individuals did not have sufficient volume, the analysis was performed on 18 individuals. In the normal activity, the mean salivary cortisol concentration (n = 18) was 8149.3 pg/mL 5 min before and 10,853 pg/mL 5 min after, where as in the excessive activity, it was 14,874 pg/mL 5 min before and 23,179 pg/mL 5 min after. Upon comparing the mean values, a significant difference was observed between the before and after values for the normal interactive activity (df = 17, t = −2.11, *p* = 0.049) and also between the before and after values for the excessive interactive activity (df = 17, t = −4.32, *p* = 0.0005). Furthermore, in both 5 min before normal and excessive and 5 min after normal and excessive, significant differences were observed also. (before: df = 17, t = 3.812, *p* = 0.0007, after: df = 17, t = −4.967, *p* = 0.0001) (Figure 8).

### 3.5. Relationship between the Changes in Body Temperature, Frequency of Negative Behavior Expression and Changes in Cortisol Concentration

The results of the relationship between the body (ear) temperature change and frequency of behavioral expression, and the change in cortisol concentration in relation to ear temperature change are shown in Table 2.

Furthermore, the following contrasting trends were observed between Freezing-expressing (n = 11) and non-expressing (n = 7: one cortisol concentration was not measured) pigs in the body temperature change and cortisol concentration change 5 min before and 5 min after activity, that is, the increase in body temperature was higher in the former (mean 0.84 °C in the former, mean 0.19 °C in the latter) and the change in cortisol concentration was higher in the latter (mean 6751 pg/mL in the former, mean 10,748 pg/mL in the latter). A negative correlation was found between the change in body temperature between the beginning and end of interactivity and the change in cortisol concentration in “Freezing”-expressing individuals (r = 0.62, t = −2.38, *p* = 0.030), whereas no correlation was observed in those who did not express “Freezing”.

## 4. Discussion

### 4.1. Body Temperature Measurement as a Monitoring Method for the State of Welfare

Body temperature has been cited as one of the effective indicators in confirming the welfare status of animals, including this species [21]. Moreover, body temperature measured from the body surface or eye temperature has been used as an alternative to rectal temperature for health management and stress assessment. For instance, this has been confirmed in cats, where a correlation was observed between rectal temperature values representing core body temperature and eye temperature [22]. In the context of stress measurement in guinea pigs, a previous study used eye temperature using thermal cameras as an indicator [9]. In the present study, body temperature measurements obtained using ear canal predictive thermometers showed a correlation between body temperature measurements and rectal temperature measurements. This indicated that temperature measurement during interactive activities is feasible. Once the measurer has acquired knowledge of the guinea pig’s external ear canal structure and the animal becomes accustomed to having the temperature sensor inserted into the ear canal, it should be possible to reduce both measurement and restraint time. This would allow for stress-free temperature measurement in this species.

### 4.2. Temperature Changes, Stress Hormones, and Behavior

Although body temperature gradually decreased to normal and gradually in-creased to excessive, BT was higher in the former than in the latter at [TP1]. One possibility is the experience of exposure to the handler. In Normal, the nervousness of being placed in the waiting area proximity to an unseen handler may have caused a rise in body temperature, while in Excessive, the animals were moved to the same waiting area a week later, which may have anticipated that they would be placed on the handler’s lap. Secondly, there may have been an effect of inserting the swab into their mouth; it was inserted again a week later at the same time, but acclimatization occurred, and there may not have been an increase in temperature.

In this experiment, a significant difference was already observed in the concentrations before the two methods of interaction, the analysis must remain within each method. Possible reasons for this are differences in the sound environment at the experiment site and differences in the number of people surrounded. The experiment was conducted inside a zoo, and both days were closed, but on the “excessive” activity environment, compared to the “normal” activity environment, there was more noise from the chirping of birds, construction noise from the facility next enclosure was echoing. In addition, the number of people who stayed at the experiment site was 7 in normal, but 10 in excessive. It suggested that these environmental differences caused the start. Nevertheless, it may be important to note that crowding with visitors and the noise associated with high numbers of users may be more stressful than the interaction activities themselves.

In terms of the relationship among behavior, body temperature, and cortisol concentration, a significant negative correlation was observed between the changes in body temperature and cortisol concentration in pigs expressing “Freezing” only during the excessive interactive activity. “Freezing” is a defensive response, immobile posture to avoid detection by predators, and is considered an expression of fear toward an unfamiliar human when the animals are bred in captivity [16]. Although female guinea pigs exhibit a decrease in core body temperature when placed in an unfamiliar environment [19], in contrast to the tendency to increase body temperature observed in freezing pigs in this study. Based on these observations, it can be hypothesized that the patterns of body temperature increase and decrease vary between animals depending on the types of negative behaviors. In a previous study in GPs’ intervention therapy, it was reported that in an environment where hiding places can be selected, freezing and hiding place use significantly increased compared to a control group with no human contact [14]; it may be important to confirm the occurrence of “Freezing” in control groups in zoos as well. Therefore, it is necessary to gather various insights regarding negative behaviors while considering the relationship between changes in body temperature and behavior expression to clarify the key points for consideration in interaction methods.

Until now, stress has been defined as a “state of threatened homeostasis or disharmony” [23], and minimizing such a state has been a practical goal in animal welfare. The cortisol concentration in the blood or saliva has been measured for various animals, including humans. Guinea pigs have also been subjected to such measurements, including monitoring of the effect of pain relief’s effect [17,18] and measuring separation-induced stress during the early stages of growth [24]. However, varying perspectives on the relationship between stress hormone measurements and behavior exist on the relationship between stress hormone measurements and behavior. For instance, an increase in fecal corticosteroid concentrations has been observed in highly active guinea pigs, raising doubts about the straightforward association between negative states and hormone levels [25]. Some studies have also emphasized the importance of considering body weight changes and long-term welfare assessments to understand the complex relationship between negative states and hormone levels [26,27,28]. Furthermore, discussions have been made about the distinctions between chronic stress states and acute, transient stress states [6]. In this context, while stress hormones are considered to be effective indicators for assessing welfare states, stress situations are involved in regulating social behavior, and conversely, or social behavior re-regulates endocrine mechanisms and influences hormone levels [29,30]. Their interpretation requires careful consideration and judgment due to the complexity of their implication.

### 4.3. Possible Influence of Age Distribution

The subjects in this study ranged in age from approximately six months to two and a half years. In general, young guinea pigs are more susceptible to stress than older ones, and it has been pointed out that having older, familiar guinea pigs in the same group brings mental peace to them [31]. In the experiment, the animals were required to stay on each handler’s lap for 20 min, which may have put more stress on young animals than those who didn’t. Currently, the common practice for petting activities involving guinea pigs is rotation of about 30 min at a time, but from the perspective of considering animal welfare to check the condition of the animals, this is an issue for future research. Therefore, we believe that it is necessary to set conditions based on an understanding of the individual’s age and social relationship with other individuals.

### 4.4. Consideration of Negative Behaviour Ethogram for Activity Participants

It supposed that “Head tossing” can be both defensive and threatening (General ethograms [32]), and can be assumed to bring negative emotions, it is important to take care that human hands do not enter the field of view and pose a threat to the GP. “Head turning” is a behavior described by care staff as “restless’’ and is often observed prior to “Locomotion”, so it is necessary to determine whether this behavior will continue to be transferred to “Locomotion” or be discontinued. Although ‘Licking’ was less common overall, care staff information indicates that grooming is frequently observed in the pen, and it often lasts for more than 10 s if not disturbed by other animals. Grooming is essentially self-maintenance and not a negative behavior. Therefore, it is necessary to redefine ‘Licking’ comparing grooming, whether the short 1–2 s of grooming is really ‘grooming’ or the animal just chooses its own fur to nibble on. It is necessary to adopt “Head tossing”, “Locomotion”, and “Freezing” as behaviors that can be easily observed by interaction participants, including children can easily observe, redefine ‘Licking’ or grooming, and to refine the ethogram of negative behaviors.

Behaviors that can be clearly counted can be recognized by participants while interacting, but measuring duration, which requires tracking for a while, seems difficult to balance with activity. Therefore, participants are encouraged to observe the animals in their lap using ethograms that are easy to count. For behaviors that require tracking, it may be desirable to observe the animal on another participant’s lap.

Furthermore, some guinea pigs have been observed to calmly close their eyes or adopt a resting posture when being gently stroked during petting activities. Developing an ethogram of these positive behaviors makes it possible to confirm welfare status from both positive and negative perspectives.

### 4.5. Possibilities and Challenges in the Promotion of Animal Visitor Interaction Procedure

In this investigation, the guinea pigs used in this experiment had not participated in any activities before, and it is expected that as the interaction activities progresses, they will become accustomed to the environment and being touched by the children. Observation and body temperature measurements are required when people participate in situations involving them. Ethically advancing the use of this species in zoos involves evaluating the animals’ experiences, including human interaction and contact, during interactive activities [33,34,35]. This aligns with the current practice of AVIP and is of significant importance. During these activities, it is desirable for staff and participants should share information about the meanings and roles of various behaviors and monitor the animals together. This infrared ear thermometer was designed for use in the ear canal of human infants and was used to attempt measurement after confirming the structural similarity between the human and guinea pig external auditory canal. Once the guinea pigs become accustomed to having the thermometer sensor inserted into their ear canal and the measurement personnel familiarize themselves with the anatomy of the guinea pig’s external auditory canal, safe temperature monitoring becomes feasible. As of 2022, approximately 90% of the 90 zoos affiliated with the Japanese Association of Zoos and Aquariums (JAZA) in Japan have guinea pigs as part of their exhibits [36], with the primary purpose being their visitor engagement. In these settings, a close observation of morphology and behavior is feasible. If a safe and noninvasive measurement method can be established for guinea pigs, safe temperature measurement can be conducted along with the participation of visitors. Furthermore, it is essential to continue studying the differences between behaviors in the animals’ everyday living environments and during interaction sessions, focusing on negative behavior and examining the relationship between positive behavior and temperature measurements or physiological indicators. In addition, individual characteristics of animals should be considered and further data should be accumulated.

### 4.6. Limitation of Research

Although the number of samples required (95% confidence interval, 10% error) for the number of individuals scheduled to participate in interaction activities at the study site 35 was 25.6, the number of subjects was less than this. Another limitation is that the guinea pigs were assigned to handlers sequentially by care staff according to the list; they were not blinded. Furthermore, there was no control group in this study, and the effects of interactive activity were not investigated; it is necessary to investigate the baseline of body temperature changes and behavioral expression under no human interactive conditions by temperature transponder/chip in the future. It should be mentioned that the results of this study should be treated as informative.

## 5. Conclusions

This study aimed to develop a method for advancing welfare assessment in guinea pig interaction activities conducted in many Japanese zoos. The results showed that ear temperature measurement could be used instead of rectal temperature as a simple temperature measurement method. Second, there was a significant decrease in body temperature before and after the recommended normal activity and an increase in body temperature during the non-recommended excessive activity, with a significant increase in the concentration of the stress hormone salivary cortisol in the latter. However, the significant relationship between the onset of negative behavior, body temperature, and stress hormone concentrations was partial. Although AVIP practice needs to work with participants on the stress state of each individual in real-time during interaction activities, more data are needed to confirm the scientific validity of this method.

Future research directions could include the following. (i) To develop a method for measuring body temperature in the ear canal that is safer and can correlate with rectal temperature, to understand diurnal changes in body temperature, and to compare body temperature when participating in interactive activities and at their enclosure; (ii) To conduct research on differences between interactions with care staff and interactions with children, looking at the relationship between frequency of action and temperature changes; (iii) To find out the difference between behavior during contact activities and in enclosure using an ethogram related to comfortable state.

## Figures and Tables

**Table 1:** Guinea pig negative behavior: definition and unit for analysis.

Ethogram	Definition	Unit of Analysis	Comparison Unit	Reasons to Be Considered “Negative”	Remarks
Head Tossing	Tossing the head	Number of tosses	Incidence rate per 60 s	As this behavior defined as aggressive behavior [16], suppossed some kind of desire for exclusion.	
Head Turning	At least one of the forelegs moved and the head turned by ≥90° in the direction of leg movement.	Number of turns	Incidence rate per 60 s	Since it is thought that the head turning is to be search the direction move, but the animal can’t find where to go.	
Locomotion	All four legs moved from their previous position. If the next movement starts within 2 s, it was defined as consecutive movement.	Number of movements	Incidence rate per 60 s	Attempts to move away from the human hand, but this behaviour is deterred by the handler or care staff with the aim of preventing a fall. Suppression of the urge to move was judged to be ‘negative’.	Locomotion is not considered a negative behaviour as a stand-alone behaviour, but movement away from other individuals as a social behaviour is considered a negative behaviour [17].
Licking	Grooming with the teeth or paws was observed.	Number of actions	Incidence rate per 60 s	Licking or gnawing a cage or floor mesh is defined as an avoidance behavior, but if there is no object, it is considered to be a compensatory behavior that lightly bites hair or paws of their own body [18,19].	This may include coprophagy. It may include behavior such as briefly nibbling fur and scratching the body with a hind leg.
Freezing	Standing or cessation at least both forelegs for approximately ≥2 s.	Total duration	Total observed duration/total observable time	Freezing occurs with a decrease in comfortable behaviors and can also be seen when the animals are in pain [15,18].	When the next episode of freezing starts within 1 s it will be recorded as consecutive.

**Table 2:** Changes in Body Temperature, Frequency of Negative Behavior Expression and Changes in Cortisol Concentration.

		Temperature Change	Cortisol Concentration Change			Temperature Change	Behaviour Expression				
Activity		Average ear temperature change between Session A start and Session B end (°C)	Average salivary cortisol concentration change between 5 min before and 5 min after	Pearson’s correlation coefficient between ear temperature change and salivary concentration change		Average ear temperature change between Session A start and Session B end (°C)	Ethogram	Mean frequency occurrence (60 s.)		Total frequency A and B	Pearson’s correlation coefficient between ear temperature change and total frequency
	Subjects				Subjects						
	
	Body weight				Body weight						
	average (g)				average (g)			Session			
								A	B		
Normal							Head tossing *	0.65	0.44	1.09	0.544
							SD	0.36	0.31		(t = 2.048, df = 10, *p* = 0.068)
							CV	0.55	0.70		
	18	−0.37	2703	0.101	12	−0.28	Head turning	0.20	0.09	0.29	−0.095
	925	(S.D. = 1.87)	(S.D. = 5144)	(t = 0.404, df = 17,	923	(S.D. = 2.00)	SD	0.36	0.11		(t = −270, df = 10, *p* = 0.793)
				*p* = 0.691)			CV	1.80	1.22		
							Locomotion	0.37	0.15	0.52	0.565
							SD	0.57	0.15		(t = 1.936, df = 10, *p* = 0.082)
							CV	1.54	1.00		
							Licking	0.14	0.32	0.46	−0.066
							SD	0.19	0.40		(t = −0.188, df = 10, *p* = 0.855)
							CV	1.36	1.25		
Excessive							Head tossing *	1.12	0.70	1.82	−0.049
							SD	0.78	0.77		(t = −0.203, df = 17, *p* = 0.842)
							CV	0.70	1.10		
	18	0.74	8304	−0.358	19	0.58	Head turning	0.42	0.35	0.77	−0.178
	925	(S.D. = 1.72)	(S.D. = 7714)	(t = 0.153, df = 17,	923	(S.D. = 1.64)	SD	0.36	0.84		(t = −0.747, df = 17, *p* = 0.466)
				*p* = 0.237)			CV	0.86	2.40		
							Locomotion	1.24	1.21	2.45	0.194
							SD	0.92	0.97		(t = 0.814, df = 17, *p* = 0.427)
							CV	0.74	0.80		
							Licking	0.08	0.13	0.21	0.338
							SD	0.12	0.21		(t = 1.480, df = 17, *p* = 0.157)
							CV	1.50	1.62		

(* Session B significantly decreased compared to A).

## Data Availability

None of the data were deposited in an official repository.
